# First Confirmed Case of Zoonotic Transmission of RR-TB from a Dog to a Human, a Neglected Mode of *Mycobacterium tuberculosis* Infection—Case Report and Review of the Literature

**DOI:** 10.3390/pathogens14070684

**Published:** 2025-07-11

**Authors:** Ljiljana Zmak, Marija Gomercic Palcic, Mihaela Obrovac, Ivana Folnozic, Drazen Strelec, Irena Reil, Ana Miljan, Maja Zdelar-Tuk, Sanja Duvnjak, Diana Mihalac, Danka Jovetic, Silvio Spicic

**Affiliations:** 1 Microbiology Division, Department for Tuberculosis, Croatian Institute of Public Health, 10000 Zagreb, Croatia; mihaela.obrovac@hzjz.hr; 2 School of Medicine, University of Zagreb, 10000 Zagreb, Croatia; marijagomercic@yahoo.com; 3 Division of Pulmology, Department of Internal Medicine, Sestre Milosrdnice University Hospital Center, 10000 Zagreb, Croatia; ivana.folnozic@gmail.com; 4 Department for Lung Diseases and Resistant TB, Service for Lung Diseases and Tuberculosis Klenovnik, Varaždin General Hospital, 42244 Klenovnik, Croatia; drazen.strelec@gmail.com; 5 Laboratory for Bacterial Zoonosis and Molecular Diagnostics of Bacterial Diseases, Department of Bacteriology and Parasitology, Croatian Veterinary Institute, 10000 Zagreb, Croatia; reil@veinst.hr (I.R.); zdelar-tuk@veinst.hr (M.Z.-T.); marjanovic@veinst.hr (S.D.); spicic@veinst.hr (S.S.); 6 Veterinary Practice “Mačji Kašalj”, 10000 Zagreb, Croatia; ana8miljan@gmail.com; 7 Epidemiology Division, Andrija Štampar Teaching Institute of Public Health, 10000 Zagreb, Croatia; diana.mihalac@stampar.hr; 8 Microbiology Division, Public Health Institute of Dubrovnik-Neretva County, 20000 Dubrovnik, Croatia; dankajovetic@yahoo.com

**Keywords:** *Mycobacterium tuberculosis*, RR/MDR-TB, zoonoses, dog–human transmission

## Abstract

*Mycobacterium (M.) tuberculosis* mostly spreads from active tuberculosis (TB) patients to human contacts, although human-to-animal and animal-to-human transmission has been described. Here, we present a rare case of rifampicin-resistant tuberculosis (RR-TB) transmission from a companion dog to its owner, highlighting the zoonotic potential of the pathogen. Namely, a 37-year-old Croatian man was diagnosed with RR-TB, with whole-genome sequencing analysis revealing a close genetic link to the strain isolated from his dog, which had died of miliary TB six years earlier. This case emphasizes the complexity of TB transmission dynamics, particularly involving companion animals, and underlines the importance of integrated “One Health” approaches for TB control. Awareness of zoonotic TB risks is essential for the early detection and prevention of cross-species transmission, especially in vulnerable populations and households with close human–animal contact.

## 1. Introduction

*M. tuberculosis* is primarily a human pathogen and is rarely described as a causative agent of tuberculosis (TB) in other animal species. Among the members of the *M. tuberculosis* complex (MTBC), the causative pathogens of TB, the ones most widely associated with zoonotic potential are *M. bovis* and *M. caprae* [1]. However, there are scarce reports of TB caused by *M. tuberculosis* in animal species known to live in close contact with humans, including elephants, dogs, cats, goats, and cattle [2]. However, in the majority of cases, the infection is transmitted from humans to animals, or animals to animals, while there are no reports of confirmed transmission of *M. tuberculosis* from animals, including dogs, to humans leading to active disease. There is scientific evidence that dogs can develop pulmonary TB, thus being potentially infectious for other animals or humans. When naive animals were exposed to experimentally infected dogs, culture confirmation of infection could be made, confirming the respiratory pathway as a possible route of infection [3]. Bonovska et al. also confirmed that dogs could be infected orally, as dogs fed with *M. tuberculosis*-contaminated meat developed active disease [3]. Interestingly, pathological changes were found in both the gastrointestinal tract and in the lungs of tested animals, making the animals a possible source of infection. The potential danger that TB infections in companion animals could pose a threat to human health was an important topic more than half a century ago, when several authors emphasized the possible role of animals as reservoirs for human TB infection. Namely, during the 1940s and 1950s, TB was found in around 4% of dog necropsies [4]. This possible zoonotic potential of *M. tuberculosis* is especially worrisome for persons at increased risk of developing active TB, including young children and immunocompromised individuals.

One of the biggest obstacles for TB elimination is the pathogen’s potential for acquiring resistance, and today, we have around 400,000 MDR/RR cases of TB yearly [5]. It is well known that poor TB therapy administration, including monotherapy or short therapy duration, could lead to resistance. Mortality and the overall burden of disease are much higher for resistant strains in comparison to TB caused by susceptible strains. In Croatia, there is a favorable situation regarding MDR/RR *M. tuberculosis* strains. In the past five years (2019–2023) in Croatia, among all culture-confirmed TB cases, we had just two MDR-TB patients, thus being well below the average percentage of TB resistance in EU/EEA countries [6]. Moreover, according to official laboratory and surveillance data, we have never had a case of TB caused by a strain monoresistant to rifampicin (data provided by the Croatian TB Reference Laboratory Resistance Registry). Here, we present a case of RR-TB in a Croatian patient without a history of previous TB treatment but with a description of contact with a dog with confirmed RR-TB.

## 2. Case Report

### 2.1. Human

**Figure 1:**
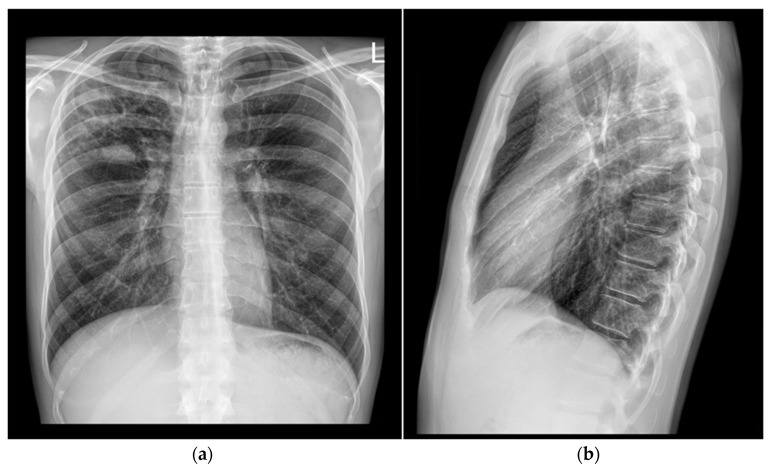
Chest X-rays (25 April 2024). In the upper lobe of the right lung, an area of inhomogeneous opacity is observed, containing a cavity measuring 27 mm in diameter. Additionally, there are fibroadhesive and reticulonodular opacities, along with a larger soft tissue mass exhibiting nodular characteristics: (**a**) frontal presentation; (**b**) lateral presentation.

A 37-year-old man was admitted to the Division of Pulmonology, Department of Internal Medicine, Sestre milosrdnice University Hospital Center, on 25 April 2024 due to a two-month history of subfebrile temperature, malaise, productive cough, and night sweats. During the same period, he had experienced loss of appetite and an unintentional weight loss of 4 kg (BMI: 21 kg/m^2^). The patient had no known chronic medical conditions. He reported occasional cigarette smoking and denied alcohol or illicit drug use. Notably, he had no contact with a known TB patient but reported that his dog had died of disseminated TB six years prior. Based on the patient’s symptoms and a chest X-ray showing features suggestive of a lung abscess in the right upper lobe, his general practitioner initiated empirical antibiotic therapy with amoxicillin/clavulanate (1000 mg t.i.d.) for seven days and referred him to a pulmonologist for further evaluation (Figure 1).

On physical examination, diminished breath sounds were noted in the right upper lobe. Laboratory investigations revealed moderate leukocytosis (10 × 10^9^/L) and normocytic anemia (erythrocytes: 3.72 × 10^12^/L; hemoglobin: 117 g/L; MCV: 91.9 fL). Biochemical parameters were within normal limits, except for a mildly elevated C-reactive protein level of 16.6 mg/L. On 26 April 2024, sputum analysis confirmed the presence of acid-fast bacilli (AFB) on smear microscopy examination and a positive Xpert MTB/RIF Ultra (Cepheid, Sunnyvale, CA, USA) assay indicating rifampicin resistance, together with a positive *M. tuberculosis* culture 12 days later. Phenotypic drug susceptibility testing using BACTEC MGIT 960 (Becton Dickinson, Franklin Lakes, NJ, USA) confirmed resistance to rifampicin. *M. tuberculosis* DNA was isolated using modified in-house protocol according to Bainomugisa et al. [7]. Sample libraries for sequencing were prepared using the Illumina DNA prep kit (Illumina Inc., San Diego, CA, USA) according to the manufacturer’s instructions. The libraries were sequenced using the MiniSeq system (Illumina Inc., San Diego, CA, USA) with an output of 2 × 151 bp paired-end reads. FASTQ files were uploaded to TB Profiler (version 6.2.2) webtool for drug resistance prediction, as well as to MTBseq pipeline (version 1.1.0) for both drug resistance prediction and phylogenomic analysis [8,9,10]. Both TB Profiler and MTBseq identified a missense variant His445Tyr in rpoB gene, a mutation that is associated with resistance according to WHO catalogue, thus confirming molecular background of resistance to rifampicin [11]. Distance analysis was performed using MTBseq, and the isolate sequence was compared to the strain isolated from the patient’s dog six years earlier. The analysis revealed an SNP distance of only four SNPs, thus confirming the transmission between the dog and the patient.

**Figure 2:**
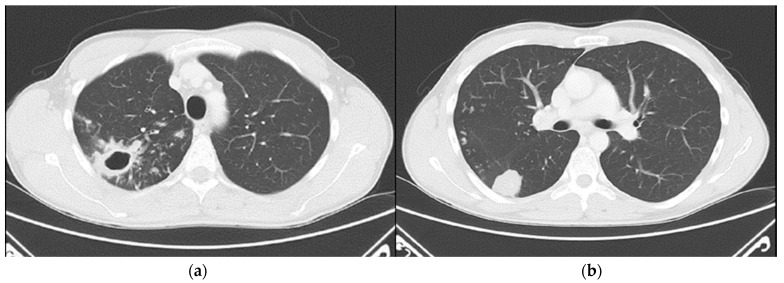
Chest CT scans (29 April 2024): (**a**) large cavitary lesion in the right upper lobe; (**b**) nodular lesion in the lower lung lobe and a discrete “tree-in-bud” pattern.

Chest computed tomography (CT) revealed numerous small confluent nodular lesions in the apical and posterior segments of the right upper lobe, forming a characteristic “tree-in-bud” pattern. These lesions ranged in size from a few millimeters to 2–3 cm and surrounded a cavitary lesion measuring up to 43 mm, adjacent to the pleura. A 3 cm nodular lesion with small calcifications was also observed in the upper segment of the right lower lobe, accompanied by a similar but less pronounced “tree-in-bud” pattern (Figure 2).

**Figure 3:**
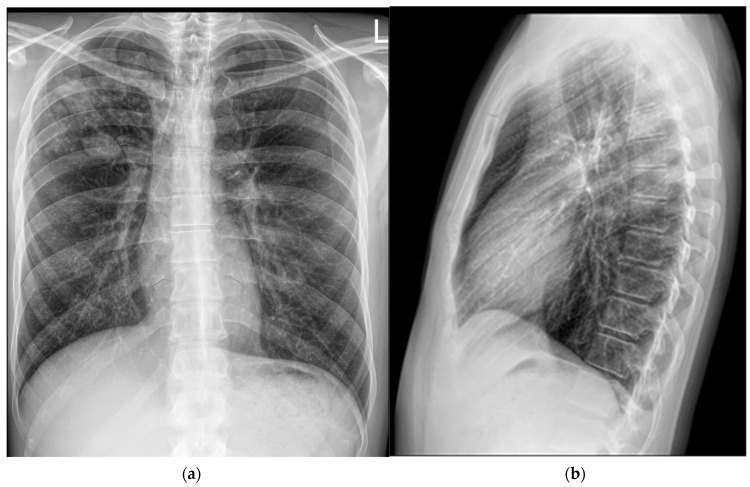
Follow-up chest X-rays (5 June 2024) show complete resolution of the previously described cavity in the right upper lobe. However, a stable soft tissue infiltrate with oval-shaped opacity persists in the same region: (**a**) frontal presentation; (**b**) lateral presentation.

Intrathoracic lymphadenopathy was present. On 30 April 2024, the patient was transferred to the Department for Lung Diseases and Drug-Resistant Tuberculosis at Klenovnik, Varaždin General Hospital, where a shortened six-month treatment regimen (bedaquiline, pretomanid, linezolid, and moxifloxacin: BPaLM) was promptly initiated [12]. This was the first patient in Croatia diagnosed with a monoresistant form of TB resistant to rifampicin who met the criteria for the aforementioned treatment regimen. As pretomanid was not registered in Croatia at the time, it was not possible to commence the full treatment regimen immediately, in accordance with the recommended treatment algorithm. The estimated time for obtaining the drug was up to three weeks. Consequently, therapy was initially started according to the BLM protocol, including bedaquiline 100 mg (four tablets daily), which the patient received until 21 May 2024, and thereafter bedaquiline was continued at a dose of 200 mg three times per week (Mondays, Wednesdays, and Fridays). Linezolid was administered at an initial dose of 1200 mg daily for 2 weeks, followed by 600 mg daily for the next 12 weeks, and then reduced to 300 mg daily for the final 8 weeks. Moxifloxacin was given at a dose of 800 mg daily during this interim period. Pretomanid treatment was initiated on 12 June 2024, at a dose of 200 mg once daily. At that time, the moxifloxacin dose was reduced to 400 mg daily, and the patient continued therapy as per the BPaLM regimen. Throughout the entire course of therapy, regular ECG monitoring was performed, with no significant QTc interval prolongation observed. Leukocyte counts remained within normal limits, with no episodes of leukopenia. The patient did not report any locomotor symptoms, and no clinical signs of polyneuropathy were detected. Control sputum samples taken during May were AFB smear-negative, while cultures from collected sputum samples became negative in June. Subsequent sputum samples—collected monthly, two per sampling (total of nine additional samples) by the end of hospitalization—remained negative for both direct smear microscopy and culture. Chest X-rays showed complete resolution of the previously described cavity in the right upper lobe, with a persistence of soft tissue infiltrate with oval-shaped opacity in the same region (Figure 3).

**Figure 4:**
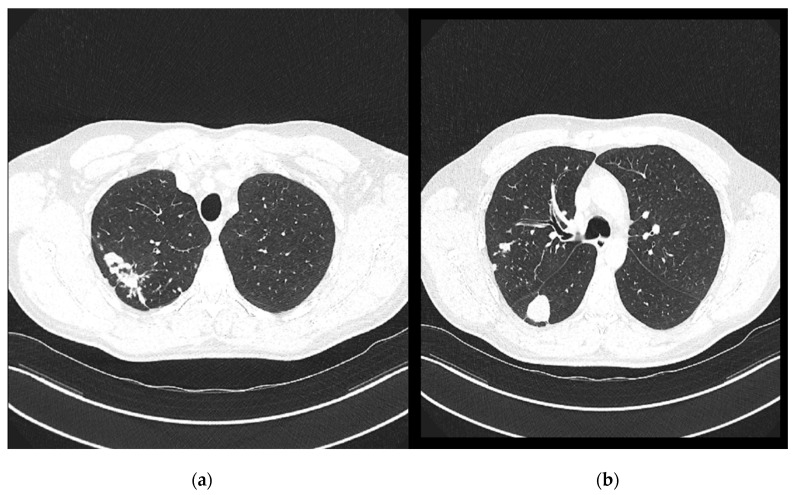
Chest CT scans (7 November 2024): (**a**) solid lesions in upper lobe; (**b**) tuberculoma in the upper segment of the right lower lobe.

On 7 November 2024, a follow-up contrast-enhanced MSCT of the thorax was performed and compared with the previous CT scan from 29 April 2024 (Figure 4). It revealed several irregular, confluent solid lesions in the right apical region, the largest measuring 23 mm, 29 mm, and 20 mm in diameter, consistent with tuberculomas. Band-like fibrotic changes extending to subpleural and perifocal areas were also noted. In addition, micronodular interstitial changes and a “tree-in-bud” pattern were present, along with several solid parenchymal nodules up to 5 mm in diameter, suggestive of miliary tuberculomas. A tuberculoma measuring 26 × 26 mm was identified in the upper segment of the right lower lobe, with subpleural localization. Only a few reactive lymph nodes remained, with the largest measuring 9 mm in diameter.

In addition to these radiological findings, the patient demonstrated clear clinical improvement. Subjective recovery was accompanied by objective evidence of significant progress, including a substantial weight gain. The patient’s body weight increased from 56 kg at admission to 84 kg at discharge—an overall gain of 28 kg—corresponding to an increase in BMI from 17.28 kg/m^2^ to 25.93 kg/m^2^. The treatment was fully completed in accordance with the prescribed protocol and was carried out entirely in an inpatient setting. At the follow-up examination in March 2025, the patient was subjectively in good general condition, without respiratory complaints, with stable body weight and clinically well, with stationary chest radiological findings. A follow-up contrast-enhanced MSCT of the thorax is planned for June 2025.

### 2.2. Canine

In 2018, a six-year-old female American Staffordshire Terrier initially presented with hematuria and fever, with indicated carcinomatosis peritonei, a suspected pancreatic tumor, and mild ascites. The symptoms progressed over time with hematemesis and unilateral nasal bleeding. Despite good appetite, she was underweight and systemically weak. Clinical examination revealed pale mucous membranes and no abdominal pain or masses. Blood tests showed leukocytosis, neutrophilia, monocytosis, and elevated total proteins, globulins, and alkaline phosphatase. Thoracic radiographs identified a mass near the sternum and pulmonary opacities; fine-needle aspiration indicated pyogranulomatous inflammation. Lung auscultation revealed diminished sounds bilaterally. Ultrasound examination confirmed an enlarged pancreas with hyperechoic lesions. The dog was hospitalized and treated with enrofloxacin, analgesics, gastroprotectants, and opioids. Blood parameters fluctuated, indicating ongoing systemic inflammation. A laparotomy was indicated, and the procedure revealed significant organ enlargement, peritoneal lesions, and abnormal liver changes. Biopsy samples were taken, and histopathology identified severe granulomatous inflammation. Despite a range of treatments, including pain management, fluid therapy, and antibiotics (including rifampicin), the dog’s condition continued to deteriorate. New symptoms, including instability in the knee, jaundice, and dark stool, were observed, signaling progressive organ involvement. Due to the animal’s rapid decline and lack of reflex response, the owner consented to euthanasia in October 2018. Laboratory examination of extirpated mesenteric lymph nodes and liver samples confirmed the presence of *M. tuberculosis*, both by culture and PCR testing (IS6110 amplification and the GenoType^®^ Mycobacterium MTBC test, Hain Lifescience, Nehren, Germany). Antimicrobial susceptibility was tested for 13 antibiotics commonly used in human medicine using the broth microdilution method by Sensititre™ MYCOTBI Plate (Thermo Fisher Scientific, Waltham, MA, USA). Susceptibility testing showed resistance to rifampicin.

After TB was confirmed, veterinary authorities notified the competent epidemiological service. An epidemiological interview with the dog owner revealed that the animal had lived in a three-member household and had been ill for several months, with notable weight loss. At that time, three other dogs were also present in the household. The owner denied any signs of tuberculosis-like illness in the other dogs and claimed that they had undergone diagnostic evaluation (chest X-rays and blood tests) in a veterinary clinic, with no indication of either acute or latent tuberculosis infection.

The three household members and two frequent visitors to the home were referred for pulmonary evaluation. All denied having any symptoms indicative of tuberculosis. In October, four contacts tested negative on IGRA testing, including the dog owner, while one household contact tested positive and underwent further diagnostic workup. Three subsequent sputum samples were negative for *M. tuberculosis* by both direct smear microscopy and culture.

## 3. Discussion and Review of the Literature

In the past 25 years in Croatia, we have had several documented cases of zoonotic transmission leading to active TB caused by different species belonging to the MTBC group, including *M. bovis* and *M. caprae*, but never of *M. tuberculosis* [13,14]. Moreover, we also had a case of *M. pinnipedii* infection in a patient presenting with pulmonary TB, but the source of infection was not found [14]. Of the species belonging to the MTBC group, the zoonotic potential of *M. bovis* is best documented, with many cases of animal-to-human transmission reported during the years, especially before the implementation of eradication campaigns, along with the pasteurization of milk. In most cases, cattle were the source of infection for humans, but the transmission of *M. bovis* from camels and white-tailed deer has also been described [15]. Zoonotic transmission caused by other species of the MTBC group, including *M. caprae*, *M. orygis*, *M. pinnipedii*, and *M. suricate*, is rare, with few case reports in the literature.

Studies have shown that various animal species, including dogs, can harbor *M. tuberculosis*, although such cases are relatively rare [16]. In a study by Pesciaroli et al., *M. tuberculosis* was reported in several animal species living in close contact with humans, including elephants, dogs, and cats [2]. Most cases involved the transmission of the disease from humans to animals, rather than the reverse.

The case presented herein is the first proven case of interspecies *M. tuberculosis* transmission from a dog to a human in Croatia confirmed by WGS analysis of both strains, human and canine. To our knowledge, this is also the first confirmed case of zoonotic transmission of *M. tuberculosis* from any animal species to a human in Croatia and the first confirmed case of RR-TB transmission from a dog to its owner worldwide. The presented case gives valuable insights into many challenges present when dealing with both human and animal TB. The diagnosis confirmation for the presented case of active TB in a six-year-old female American Staffordshire Terrier was performed only after autopsy, highlighting the lack of experience that veterinarians have with TB. However, this is somehow expected considering the rare occurrence of the disease in dogs globally. In addition, the clinical signs in dogs with TB are non-specific and the diagnosis is frequently delayed. Nonetheless, even if the diagnosis is made early in the course of the disease, treatment of TB in dogs is discouraged, due to the expected poor clinical outcome. There is no standard TB therapy recommended for dogs, and while some human anti-TB drugs like streptomycin are not even approved for administration in animals, others, like isoniazid and rifampicin, usually cause severe side effects, including neurological problems and hepatotoxicity. When considering therapy in dogs with active TB, we should also take into consideration the possibility of TB transmission to both human and animal contacts from the affected dog. Dogs with active TB can spread the bacilli via different routes, including respiratory secretion, saliva, urine, and feces [17]. The transmission of *M. tuberculosis* from experimentally infected dogs to naive animals has been demonstrated, suggesting that dogs can shed mycobacteria in the environment and potentially act as a source of infection for other animals, including humans [3]. This dog was in close contact with family members and other dogs living in the household for several months before the owners agreed to euthanize their pet. Epidemiological investigation revealed that the dog had no evident close contact with individuals suffering from active TB, leaving the exact source of infection for the reported dog unknown. Therefore, the dog likely contracted the infection through incidental exposure to an infected person or infectious excreta. The route of transmission may have been the inhalation of aerosols from a patient with active TB, but given the involvement of internal organs and the gastrointestinal symptoms observed in the dog, the ingestion of contaminated sputum or contaminated food appears to be a more plausible pathway. Our findings counter the conventional belief that a long and close contact between humans and animals is needed for TB transmission. Studies have shown that nearly half of dogs exposed to patients with active tuberculosis exhibit evidence of infection, yet the development of active disease is uncommon [18]. While the risk of dog-to-dog transmission of TB is considered higher than the risk of dog-to-human transmission, no other dogs in the household had any signs of TB infection or signs of disease.

The occurrence of active TB in dogs caused by *M. tuberculosis* is extremely rare, with just several cases reported worldwide in the past 25 years. In a case report from Brazil, a Beagle was probably infected and developed active TB by ingesting contaminated food prepared by its owner, who had active TB, while in Tennessee, a 71-year-old female patient infected her 3-year-old male Yorkshire Terrier [17,19]. In Germany, a Golden Retriever with respiratory symptoms and in close contact with its owner, who had active cavitary TB, was found to have *M. tuberculosis* infection [20]. Moreover, in France, a Boxer dog imported from West Africa was diagnosed with disseminated pleural tuberculosis [21]. Here, the probable source of infection for the dog was a family cook with TB-like symptoms. In addition, one stray Maltese crossbreed dog with extensive multifocal pulmonary TB was diagnosed with *M. tuberculosis* infection in South Africa, while in California, a Golden Retriever with respiratory symptoms was found to have TB [22,23]. In both cases, the source of infection was not found. Interestingly, one case report from Greece including a male mixed-breed dog with acute diarrhea, inappetence, weight loss, and lethargy which was also found to have *M. tuberculosis* infection is one of the rare descriptions of a favorable treatment outcome in dogs with TB [24]. It is worth noting that the authors partially explained their decision to treat the dog despite the potential for infection transmission as no confirmed case of dog-to-human transmission had been reported. In Finland, active TB was diagnosed in a family dog imported from Romania to Finland, highlighting the possible importation of zoonotic TB cases from countries with high TB incidence [25]. While this review includes confirmed cases of *M. tuberculosis* infection in dogs from various countries, it is possible that additional cases, particularly from developing regions with limited diagnostic capacity or ones underreported in non-indexed local journals, have not been captured. This may result in an underestimation of the true zoonotic potential of *M. tuberculosis* in companion animals.

While cases of human-to-dog transmission are rare, to our knowledge, there is no documented case of dog-to-human transmission of *M. tuberculosis*, thus making our case the first confirmed case of zoonotic *M. tuberculosis* transmission from a dog to a human. In addition, as the dog was treated with rifampicin monotherapy, the strain likely became resistant to this very important TB drug. The owner developed active TB six years after the dog was euthanized, emphasizing the complexity of monitoring TB disease transmission. As WGS analysis found just four SNPs’ distance between the dog’s strain and the owner’s strain, and taking into consideration that in the past 35 years we had not had any cases of *M. tuberculosis* monoresistant to rifampicin in Croatia, we are quite certain that the dog infected its owner with this resistant TB strain. The emergence of RR/MDR-TB represents one of the greatest challenges in global TB control. The potential of *M. tuberculosis* to develop secondary resistance after the administration of anti-TB drugs highlights the importance of the careful administration of those compounds not only in humans, but also in animals. Although the owner was IGRA-negative at that time, the testing was performed just days after the dog was euthanized. We can assume that the infection of the owner occurred in the last phase of the dog’s disease, making the IGRA testing false negative due to the window period, which should be taken into consideration during contact screening and result interpretation.

## 4. Conclusions

This case report of RR-TB transmission from a dog to its owner serves as a reminder that TB is not only a human disease, but also a zoonotic threat that can spread between humans and animals. Our paper highlights the complexity of TB transmission and the challenges in controlling drug-resistant strains. While the reverse transmission of TB from animals to humans remains rare, it is a potential risk that must be taken seriously, particularly for vulnerable populations. The case also emphasizes the importance of prompt diagnosis, appropriate treatment, and the need for a comprehensive approach to TB control that includes both human and animal health considerations.

## Data Availability

FastQ files were submitted to ENA browser under sample accession SAMEA117819600.

## Abbreviations

The following abbreviations are used in this manuscript:

IGRA	Interferon-Gamma Release Assay
RR-TB	Rifampicin-Resistant Tuberculosis
MTBC	*Mycobacterium tuberculosis* Complex
MDR	Multidrug-Resistant
EU/EEA	European Union/European Economic Area
AFB	Acid-Fast Bacilli
USA	United States of America
DNA	Deoxyribonucleic Acid
FASTQ	FAST (Fast Analysis of Sequence Tags) + Q (Quality) format
WHO	World Health Organization
CT	Computed Tomography
BPaLM	Bedaquiline, Pretomanid, Linezolid, and Moxifloxacin
ECG	Electrocardiogram
MSCT	Multislice Computed Tomography
BMI	Body Mass Index
PCR	Polymerase Chain Reaction
WGS	Whole-Genome Sequencing
ENA	European Nucleotide Archive
